# Type-specific oncogenic human papillomavirus infection in high grade cervical disease in New Zealand

**DOI:** 10.1186/1471-2334-13-114

**Published:** 2013-03-03

**Authors:** Leonardo M Simonella, Hazel Lewis, Megan Smith, Harold Neal, Collette Bromhead, Karen Canfell

**Affiliations:** 1 Cancer Epidemiology Research Unit, Cancer Council NSW 153 Dowling Street, Woolloomooloo, Australia; 2 Present address: Saw Swee Hock School of Public Health, National University of Singapore, 6 Medical Drive, Block MD3, Level 3, Singapore 117597, Singapore; 3 National Cervical Screening Programme, Ministry of Health, 133 Molesworth Street, Thorndon, Wellington, New Zealand; 4 Lowy Cancer Research Centre, The University of NSW, Sydney, NSW 2052, Australia; 5 Molecular Biology, Aotea Pathology, CMC Building 89 Courtenay Place, Wellington, New Zealand; 6 School of Public Health, Sydney Medical School, The University of Sydney, Sydney, NSW, Australia

## Abstract

**Background:**

The national Human Papillomavirus (HPV) Immunisation Programme in New Zealand was introduced in 2008, and involves routine vaccination of girls 12–13 years with a catch-up for females aged up to 19 years. The aims of this study were to measure the pre-vaccination prevalence of oncogenic HPV infection in women aged 20–69 years who were participating in the New Zealand National Cervical Screening Programme (NZ-NCSP) and who were: (1) referred with high grade cytology with a subsequent histologically-confirmed high grade cervical intraepithelial neoplasia (CIN2/3) or adenocarcinoma *in situ* (AIS); or (2) were in the wider group of women who had a cytological prediction of high grade squamous disease or glandular abnormality (ASC-H/ HSIL+/AGC/AIS).

**Methods:**

Women aged 20–69 years appearing on the NZ-NCSP register between August 2009-February 2011 with a cytology record of ASC-H/HSIL+/AGC/AIS were invited to participate in the study. Liquid-based cytology specimens were tested for 37 HPV types using Linear Array genotyping. The prevalence of type-specific HPV infection was reported within women with histologically-confirmed CIN 2/3 and within the wider group with ASC-H/HSIL+/AGC/AIS cytology. Age-specific trends for the relative proportion of HPV 16/18 vs. other oncogenic types in CIN2/3 were assessed.

**Results:**

A total of 594 women with ASC-H/HSIL+/AGC/AIS cytology and a valid HPV test were recruited; of these 356 (60%) had confirmed CIN2/3 and 6 (1%) had confirmed AIS or glandular dysplasia. Positivity rates for any oncogenic HPV infection and for HPV16 and/or 18 within confirmed CIN2/3-AIS were 95% (95%CI: 92-97%) and 60% (54-65%) respectively; in all women with ASC-H/HSIL+/AGC/AIS cytology it was 87% (84-89%) and 53% (49-57%), respectively. The most common reported HPV types in women with CIN 2/3 were 16 (51%), 52 (19%), 31 (17%), 33 (13%) and 18 (12%). A trend for higher rates of HPV 16/18 infection compared to other oncogenic types was observed in younger women (p=0.0006).

**Conclusions:**

The prevalence of HPV 16/18 in confirmed high grade disease in New Zealand is comparable to that observed in Australia and European countries. Test positivity rates for type 52 appear higher than in comparable studies in other developed countries. A greater proportion of high grade lesions in younger women appear to be associated with HPV 16/18 infection.

## Background

Infection with the oncogenic types of human papillomavirus has been associated with a higher cumulative risk over time of developing histologically-confirmed high grade cervical precancerous disease (defined as cervical intraepithelial neoplasia grade 2/3 [CIN2/3] or adenocarcinoma in situ [AIS]) [1,2]. A recently updated worldwide meta-analysis of oncogenic HPV prevalence in CIN3 reported that the most common types were 16(58.2%), followed by 31(11.1%), 52(10.2%), 33(9.1%) then 58(9.0%) [3]. However, there appear to be significant regional variations - for example, in Asia the estimated prevalence of HPV 16 in high grade disease has been reported as 37.9%, whereas for the North America it is 56.8% [4].

Given the geographic proximity of Australia to New Zealand, a previously used working assumption has been that the two countries have similar HPV infection patterns [5]. In practice, however, no national data on HPV prevalence have been reported for New Zealand; and potential differences may exist because the ethnic composition in each of the countries are somewhat dissimilar, such that in the 2011 Australian Census 2.5% of respondents identified themselves as Aboriginal and/or Torres Strait Islander, whereas in the 2006 New Zealand Census 14.6% identified themselves as Maori [6,7]. In Australia, estimates of the type-specific prevalence of oncogenic HPV have been reported in cervical cancer, [8] high grade disease, [9,10] and normal cytology; [11] but none of these measures have been previously reported for New Zealand.

For countries implementing HPV vaccination programs, a baseline measure of oncogenic HPV infection in high grade disease provides the capacity to estimate the potential burden of precancerous disease which may be avertable via vaccination. Ongoing surveillance will then provide an opportunity to monitor vaccine effectiveness. In September 2008, New Zealand commenced implementation of a national HPV immunization program using the Gardasil™ vaccine (Merck, Whitehouse Station, NJ, USA) [12]. The vaccine has been shown to confer high levels of protection against new infections with HPV types 6, 11, 16 and 18 in women naåve for those types [13,14]. Vaccine delivery in New Zealand is ongoing for female cohorts aged 12–13 years, and girls and young women born from 1 January 1990 (aged 18 years or younger at the start of the program) are eligible to participate in a catch-up program up to their 20th birthday. The vaccine was available through participating schools or from family doctors, local health centres and some Family Planning clinics [12].

The primary aims of the current study were to provide a baseline measure of oncogenic HPV infection in women aged 20–69 years among: (1) a potentially ‘enriched’ population of women with high grade cervical lesions, defined as a those referred with a high grade cytology report *and* who had a histologically-confirmed high grade CIN lesion or adenocarcinoma *in situ*; and (2) among all women with a cytological prediction of high grade squamous disease or glandular abnormality (ASC-H/HSIL+/AGC/AIS) who were participating in the New Zealand National Cervical Screening Programme (NZ-NCSP). The secondary aim of the analysis was to compare oncogenic HPV prevalence in women with high grade disease in New Zealand with prior estimates from Australia and from other regions.

## Methods

### Study design and recruitment

We used a cross-sectional design for the study. Eligible participants, defined as women aged 20–69 years with a high grade cytology result notified to the New Zealand National Cervical Screening Programme Register (NCSP-R) between August 2009 and February 2011, were invited to participate. Established in 1990, the NCSP-R is a country-wide national register that is used to monitor New Zealand’s cervical screening participation, compliance with recommended management, laboratory and colposcopy standards and cervical abnormality rates. The representativeness of the NCSP-R of the cervical screening population is underpinned by legislation that requires all cervical specimens in New Zealand to be sent to the NCSP-R [15]. The register holds information on each woman including their national unique health identifier, name, address, date of birth, ethnicity, and cervical screening history. Women are able to withdraw from the register if they choose, but only a small proportion choose to do so - during the latter half of 2009 only 0.003% of women withdrew [15]. Cytology in New Zealand is classified according to the Bethesda 2001 New Zealand Modified Cytology Classification System (2005) [16]. A high grade cytology report is defined as a classification of high grade squamous intra-epithelial lesion (HSIL), atypical squamous cytology where high grade abnormalities cannot be excluded (ASC-H), abnormal glandular lesions (AGC, AIS), or cytology suggestive of invasion (squamous cell carcinoma [SC], adenocarcinoma involving endocervix [AC1], endometrium [AC2], extrauterine [AC3], adenocarcinoma alone [AC4]; malignant neoplasm [AC5]).

Women on the NSCP-R with a notified high grade cytology result were invited to participate in the study. A recruitment flowchart is provided in Figures A1 and A2 in the Additional file 1: Appendix. Contact with eligible participants was made after they were informed by their smear-taker of their high grade cytology result. Women were excluded if they were pregnant, had a health condition preventing participation in data collection, had a cervical smear suspicious of cancer, or had had a total hysterectomy (women with a total hysterectomy may have undergone cervical screening previously. These cases of hysterectomy are recorded on the NCSP-R). Eligible participants were contacted by telephone by a trained study recruitment officer located at the NCSP. Eligible women who consented to participate were asked to provide a cervical sample for HPV testing during colposcopy or have residual cytological material from their referral high grade smear tested for HPV within 4 weeks of the date that the cytology sample had been collected. Because a small proportion of participants were expected to have been in the target age group for HPV vaccination, women were also asked to self-report whether they had been vaccinated.

Follow-up with colposcopy with or without biopsy proceeded accorded to standard clinical referral protocols. For each woman, the most severe disease ranking in the SNOMED diagnostic (morphology M) category observed over the 6 month period following the high grade cytology report was used to classify histology grade. Because the current study included only women referred with a high grade cytological result, the sub-group with confirmed high grade disease can be considered an ‘enriched’ population with respect to other studies of HPV in high grade CIN, since the majority of other studies have enrolled women referred with a smear showing any grade of abnormality.

### Sample collection and processing

Depending on the usual practice at each collection site, either SurePath™ (Becton Dickinson, Franklin Lakes, NJ, USA) or ThinPrep™ (Hologic, Malborough, MA, USA) liquid-based cytology (LBC) collection systems were used. Both cytology collection systems have been validated for use for HPV detection using Linear Array® HPV Genotyping Test (Roche, Pleasanton, CA, USA) [17]. LBC samples were processed according to previously described methods [18]. Briefly, the process involved 1 mL of material aliquoted and pelleted for centrifugation at 13,000 r.p.m. Cell pellets were then re-suspended into 200μl of sterile phosphate-buffered saline. DNA was subsequently extracted using the automated MagNA Pure LC with the DNA-I extraction kit on the high performance protocol (Roche Diagnostics, Auckland, New Zealand). Once extracted, DNA material was stored at 4 degrees Celsius until genotyping was performed within 24 hours.

### HPV genotyping

The Linear Array genotyping system uses polymerase chain reaction (PCR) amplification of DNA, followed by a reverse line blot hybridization assay to detect amplified DNA products (amplicons). The reaction volume consists of 50μl of Linear Array-HPV master mix and 50 μl of DNA. The PCR amplification reaction involved an initial activation step at 95°C for 9 minutes, followed by 40 cycles of denaturation at 95°C for 30 seconds) annealing at 55°C for 1 minute, extension at 72°C for 1 minute, with a final extension at 72°C for 5 minutes [19]. The reaction uses PGMY 09/11 primers to detect HPV types. A total of 37 types of HPV are detectable (6, 11, 16, 18, 26, 31, 33, 35, 39, 40, 42, 45, 51, 52, 53, 54, 55, 56, 58, 59, 61, 62, 64, 66, 67, 68, 69, 70, 71, 72, 73, 81, 82, 83, 84, IS39 and CP6108) [20]. Type-specific oligonucleotide probes were used to differentiate hybridised HPV types. A test for the β-globin gene was used to determine specimen adequacy and PCR inhibition. Samples without β-globin were re-tested where possible a second time and rendered invalid if β-globin DNA was not able to be amplified. Detection of HPV type 52 was based on a probe which is also known to signal in the presence of HPV types 33, 35 and 58. Therefore, samples positive for HPV type 52 only contributed to the calculated prevalence of HPV 52 if they were negative for all of the potentially cross-reacting HPV types. Similar methods have been adopted elsewhere [17].

We classified HPV types according to the International Agency for Research on Cancer 2009 assessment of biological agents [21]. HPV types that were classified as oncogenic were 16, 18, 31, 33, 35, 39, 45, 51, 52, 56, 58, 59 (group 1 carcinogens; sufficient evidence for cervical cancer) and 68 (a group 2A carcinogen; limited evidence in humans with strong mechanistic evidence for cervical cancer).

### Data analysis

The representativeness of the study sample in relation to all women in New Zealand with a high grade cytology report was assessed by comparing age and ethnicity distribution with data from the NCSP for all women with a high grade cytology report [22]. The prevalence (and 95% confidence intervals) of oncogenic HPV was then assessed in all women with a high grade cytology report, in women with confirmed CIN 2 or CIN 3 (CIN 2/3), and for all women with CIN 2 or worse (CIN2+). A chi-square test for trend by age was applied to the relative proportion of the prevalence of grouped oncogenic HPV types (group 1: type 16 and/or18; group 2: other high risk types not 16 or18 [OHR]; group 3: any oncogenic type) separately in CIN2, CIN 3, and CIN 2/3 combined. For this analysis, participants were categorized into one of three age categories: 20–29, 30–39, and 40–69 years.

### Comparison with other countries

The prevalence of oncogenic HPV observed in confirmed CIN 2+ in New Zealand was compared with the findings of a prior study conducted in Melbourne [9]. This study recruited 1,679 clinic attendees at colposcopy clinics, most of whom (97%) were attending following an abnormal cytological result. HPV typing was performed using Roche Linear Array on DNA extracted from PreservCyt-stored specimens using the MagNA Pure LC system. The Australian study used an in-house primer to detect HPV type 52 in the subset of infections where the standard PGMY09/11 primers were also positive for HPV types 33, 35 and 58 [23]. The chi-square test was used to assess any differences in the reported prevalence of oncogenic HPV in confirmed CIN2+ between the two countries.

In addition, study findings for the type-specific prevalence of oncogenic HPV in histological CIN 2/3 (excluding AIS) were compared with results for histologically-confirmed CIN derived from a worldwide meta-analysis of HPV prevalence in high grade disease [24]. The original meta-analysis included both cytologically defined high grade lesions and histologically confirmed disease; but for the current comparison a sample-weighted prevalence of confirmed CIN2/3 was calculated for each reported HPV type for each region after excluding cytology-defined disease, using similar methods as those reported for the original results [24]. The regions for which a weighted prevalence was re-calculated were Asia, Europe, North America, and South/Central America. An updated meta-analysis on the worldwide distribution of HPV prevalence has also been published [3] but direct comparisons with those results could not be made here because geographic summaries of type-specific prevalence used combined cytology and histology classifications for high grade disease.

### Analysis software and ethical approvals

Analyses were undertaken using SAS V9.2 statistical software (Cary, NC, USA). Ethical approval for the study was obtained from the National New Zealand Ethics Committee, Cancer Council NSW Human Research Ethics Committee, Australia, and the University of Sydney Human Research Ethics Committee, Australia.

## Results

### Study population

**Figure 1:**
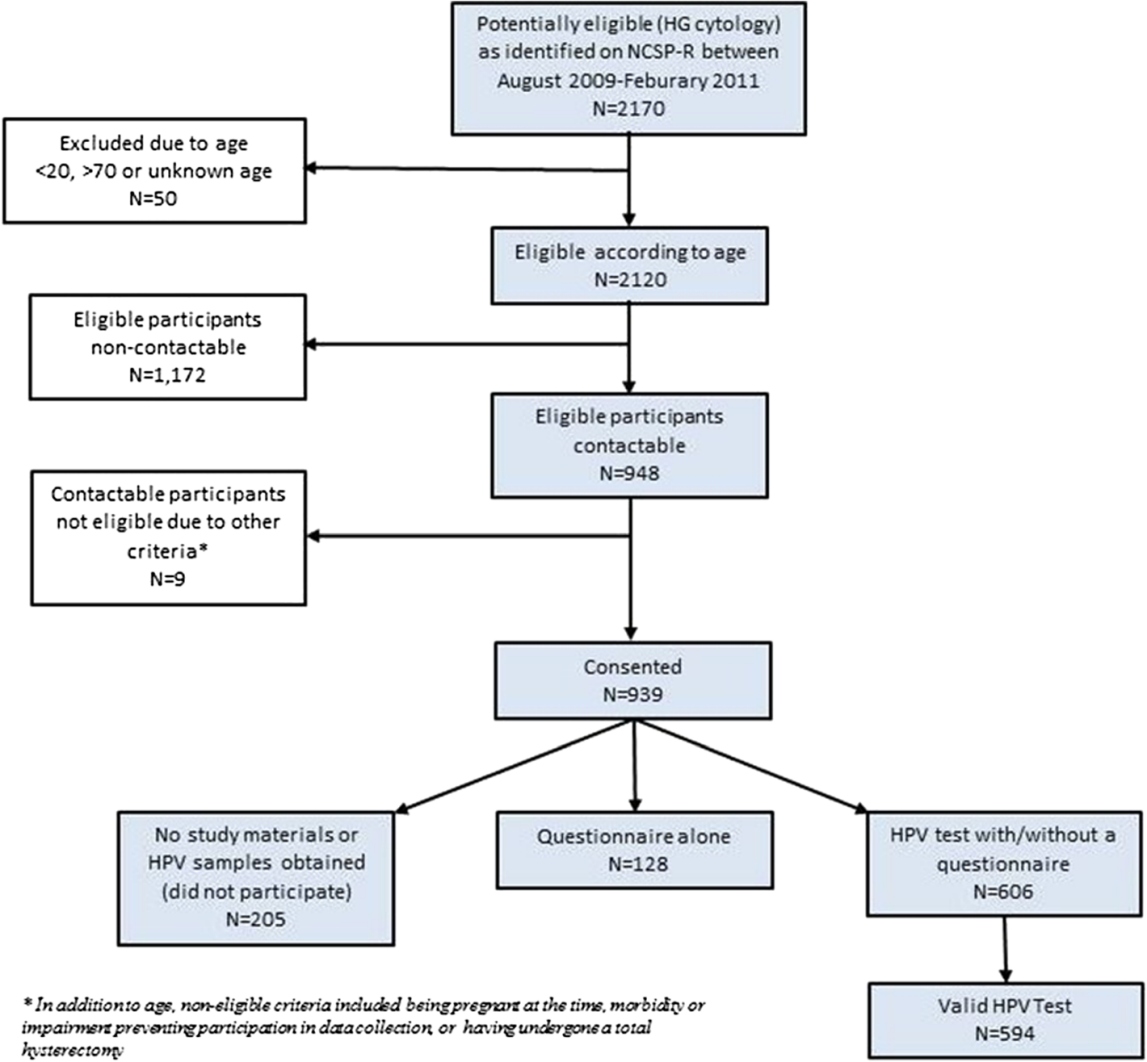
Participation in the study.

A total of 2170 women were notified to the NCSP-R as having a high grade cytology report (ASC-H/HSIL+/AGC/AIS) over the recruitment period (Figure 1). Of these, 948 were able to be contacted and were identified as eligible to participate in the study (most non-participants could not be contacted in time to obtain an HPV test sample at colposcopy, or to request permission to use the residual cytology material). Of these, a total of 594 (63%) consented to participate and provided a sample for HPV testing with a valid HPV test result. Overall, 27% of the original population identified as potentially eligible were included in the final analysis, after taking into account exclusions, ability to contact women, consent and HPV test success rates. Among the women with high grade cytology and a valid HPV test, 529 (89%) also had a valid histology result. Of these, 61% were diagnosed with a histologically-confirmed CIN 2, CIN3, AIS or glandular dysplasia, 2.2% were diagnosed with invasive cervical cancer (including a small proportion with metastatic disease) and 25.9% had confirmed low grade lesions or a negative finding on colposcopy and/or histology (Table 1).

**Table 1:** Final histological diagnosis for study participants

**Histology result**	**N**	**%**
***Histologically-confirmed CIN 2+***	***375***	***63***
**Cancer**	**13**	**2.2**
Cervical cancer - primary*	11	1.9
Cervical cancer - (metastatic disease)	2	0.3
**High grade lesion (CIN 2/3 – including AIS)**	**362**	**60.9**
CIN 3	204	34.3
CIN 2	152	25.6
AIS or glandular dysplasia	6	1.0
***<CIN 2+***	***219***	***37***
**Low grade lesion**	**88**	**14.8**
CIN 1	63	10.6
Other low grade abnormality**	25	4.2
**Negative**	**66**	**11.1**
Other non-significant abnormality^†^	38	6.4
Negative/normal	28	4.7
**No biopsy taken/reported**	**61**	**10.3**
**Insufficient for diagnosis**	**4**	**0.7**
**Total sample size**	**594**	**100%**

CIN cervical intra-epithelial neoplasia; AIS adenocarcinoma in situ.

* Invasive adenocarcinoma, invasive squamous cell carcinoma, microinvasive squamous cell carcinoma, other primary epithelial malignancy and adenosquamous carcinoma.

** Histological appearance infection with human papillomavirus (HPV), Condyloma acuminatum, Dysplasia/CIN not otherwise specified (NOS).

†Inflammation, squamousmetaplasia, polyp, other.

Of the women for whom an HPV test result was available, approximately 45% were aged between 20–29 years, and the remainder were aged between 30–69 years. A total of 16.5% identified themselves as Māori, 2.4% as Pacific peoples, 2.7% as Asian, and 78.5% as European or “other” (Table 2). Overall, the study sample had a similar age and ethnicity distribution to the broader population of women in New Zealand with a high grade cytology report (Table 2) [15]. Only 7 women (~1%) reported that they had received the HPV vaccine.

**Table 2:** High grade cervical smear cytology report by age and ethnicity among study participants and among women on the National Cervical Screening Programme-Register (NCSP-R) in New Zealand* [15]

	**Study population participants**		**NCSP-R∫**	
	**n**	**%**	**N (Jan-Jun 2009)**	**%**
**Age groups**				
20-24	148	24.9	514	22.9
25-29	136	22.9	479	21.4
30-34	92	15.5	348	15.5
35-39	79	13.3	279	12.4
40-44	52	8.8	179	8.0
45-49	34	5.7	165	7.4
50-54	25	4.2	102	4.6
55-59	17	2.9	69	3.1
60-64	7	1.2	73	3.3
65-69	4	0.7	34	1.5
**Ethnicity groups**				
European/Other	466	78.5	1678	74.9
Maori	98	16.5	367	16.4
Asian	16	2.7	127	5.7
Pacific	14	2.4	70	3.1
**Total**	**594**	**100**	**2242**	**100**

* The New Zealand NSCP-R contains cervical cytology results for all women in New Zealand, with the exception of those who choose to opt-off . For the study, and on the NCSP-R, a high grade cytology report includes women with a reported cytology result of either high grade squamous intra-epithelial lesion (HSIL), atypical squamous cells, cannot rule out a high grade lesion (ASC-H), atypical glandular cells(AGC), adenocarcinoma in situ (AIS) or cytology suggestive of invasive cancer (SC, AC1-AC5).

*∫ Screened women with high grade cytology as reported in New Zealand for the period 1st January-30th June 2009*[15]*.*

### Prevalence of oncogenic HPV

Oncogenic HPV was detected in 86.7% (n=515) of 594 women with a high grade cytology report and a valid HPV test (Table 3). This proportion increased to 94.9% when the study population was restricted to women with a histologically-confirmed diagnosis of CIN2+ (n= 375). Among women with a high grade cytology report and a valid HPV test, the most common HPV types detected were HPV 16 (44.1%), followed by HPV 52 (16.8%) and HPV 31 (15.2%). In the sub-group with confirmed disease (CIN 2+) the prevalence of these types were 51.2%, 18.9% and 17.1%, respectively (Table 3).

**Table 3:** Type specific prevalence of oncogenic HPV infection in women with (1) ASC-H/HSIL cytology^*^, (2) histologically-confirmed grade CIN 2 and (3) ≥ CIN 3

	ASC-H/HSIL cytology*^† ^**(N=594)**			**CIN 2**^**† **^**(N=152)**			**≥ CIN 3**^**†‡ **^**(N=223)**		
**HR HPV type**	**n**	**%**	**95% CI**	**n**	**%**	**95% CI**	**n**	**%**	**95% CI**
16	262	44.1	(40.1 - 48.2)	70	46.1	(37.9 - 54.3)	122	54.7	(47.9 - 61.4)
52 ^**β**^	100	16.8	(13.9 - 20.1)	24	15.8	(10.4 - 22.6)	47	21.1	(15.9 - 27.0)
31	90	15.2	(12.4 - 18.3)	37	24.3	(17.8 - 32.0)	27	12.1	(8.1 - 17.1)
33	70	11.8	(9.3 - 14.7)	26	17.1	(11.5 - 24.0)	21	9.4	(5.9 - 14.0)
18	67	11.3	(8.8 - 14.1)	16	10.5	(6.1 - 16.5)	32	14.3	(10.0 - 19.6)
58	60	10.1	(7.8 - 12.8)	21	13.8	(8.8 - 20.3)	21	9.4	(5.9 - 14.0)
51	54	9.1	(6.9 - 11.7)	12	7.9	(4.1 - 13.4)	24	10.8	(7.0 - 15.6)
39	39	6.6	(4.7 - 8.9)	14	9.2	(5.1 - 15.0)	12	5.4	(2.8 - 9.2)
45	29	4.9	(3.3 - 6.9)	4	2.6	(0.7 - 6.6)	13	5.8	(3.1 - 9.8)
59	26	4.4	(2.9 - 6.3)	5	3.3	(1.1 - 7.5)	12	5.4	(2.8 - 9.2)
35	25	4.2	(2.7 - 6.2)	12	7.9	(4.1 - 13.4)	5	2.2	(0.7 - 5.2)
56	20	3.4	(2.1 - 5.2)	3	2.0	(0.4 - 5.7)	7	3.1	(1.3 - 6.4)
68	14	2.4	(1.3 - 3.9)	4	2.6	(0.7 - 6.6)	4	1.8	(0.5 - 4.5)
16 and/or 18	314	52.9	(48.8 - 56.9)	80	52.6	(44.4 - 60.8)	148	66.4	(59.8 - 72.5)
16 and/or 18 (alone)	112	18.9	(15.8 - 22.2)	33	21.7	(15.4 - 29.1)	49	22.0	(16.7 - 30.0)
OHR	201	33.8	(30.0 - 37.8)	65	42.8	(34.8 - 51.0)	63	28.3	(22.3 - 34.6)
Single HR HPV	277	46.7	(42.6 - 50.7)	70	46.1	(37.9 - 54.3)	117	52.5	(45.7 - 59.2)
Any HR HPV	515	86.7	(83.7 - 89.3)	145	95.4	(90.7 - 98.1)	211	94.6	(90.1 - 97.2)

* High grade cytology includes high grade squamous intra-epithelial lesions (HSIL), atypical squamous cells, cannot rule out a high grade lesion (ASC-H), atypical glandular cells (AGC), adenocarcinoma in situ (AIS) or invasive cancer.

† Restricted to women with a valid HPV (human papillomavirus) test result.

‡ Includes histologically confirmed CIN 3, AIS, glandular dysplasia or cervical cancer.

^***β***^*Test positive for infection with HPV type 52 alone; not validated with linear array testing (see text).*

**Figure 2:**
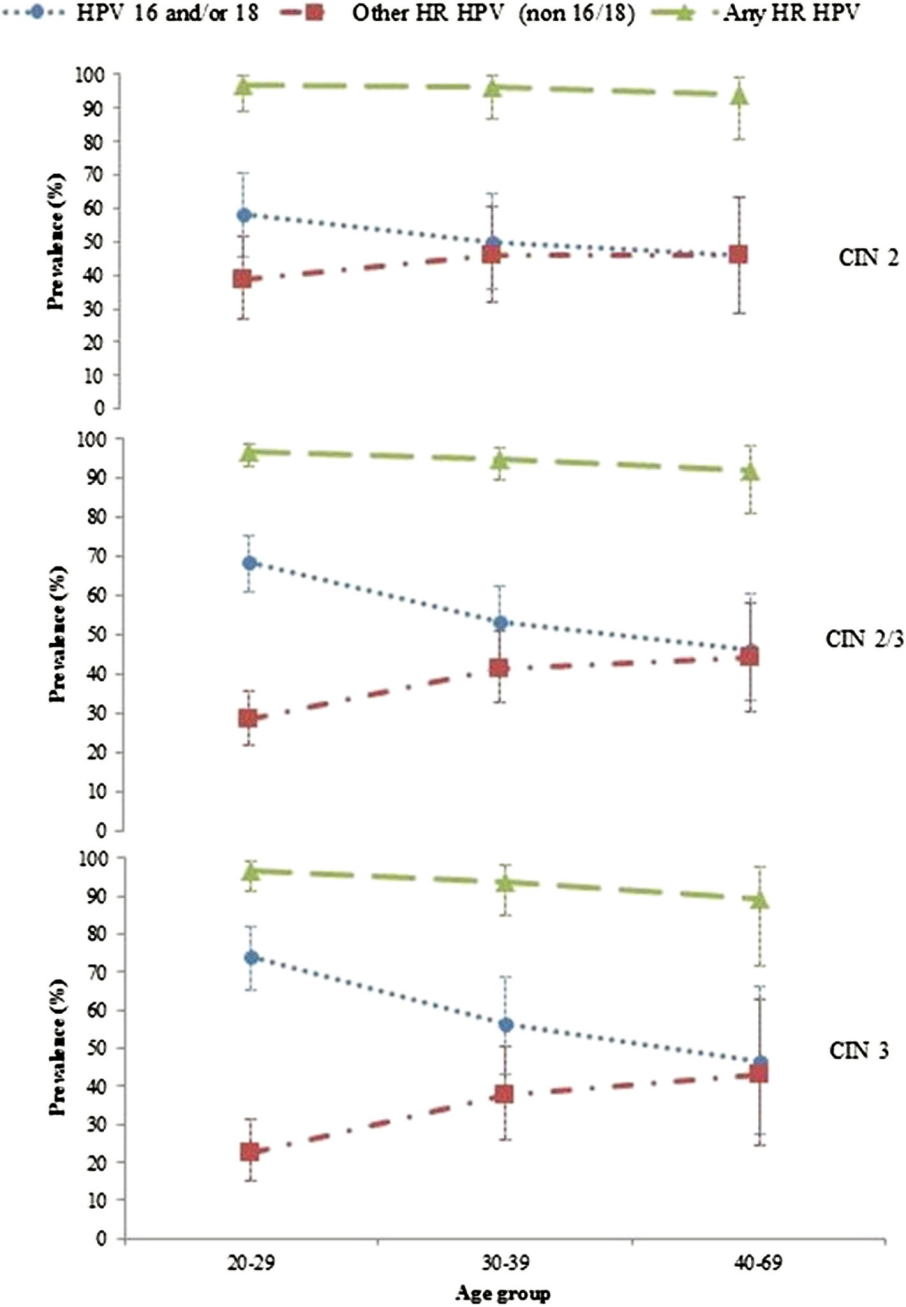
Age-specific prevalence of grouped oncogenic HPV types by histology grade (baseline estimate and 95% confidence intervals)^*^.

The combined prevalence of oncogenic HPV types 16 and 18 was 52.9% among women with a high grade cytology report, 52.6% in women with histologically-confirmed CIN 2, and 66.4% in confirmed CIN3; whereas for other oncogenic types it was 33.8%, 42.8% and 28.3%, respectively (Table 3). A significant inverse age-specific trend was observed in the relative prevalence of types 16/18 versus other HR types in women with histologically-confirmed CIN 2/3 and in CIN 3 (Figure 2). The highest relative prevalence of HPV 16/18 was observed among women aged 20–29 years (CIN 2/3: trend p=0.0006; CIN 3: trend p=0.002), whereas the highest relative prevalence of OHR types was observed in women aged 40–69 years (CIN 2/3 trend p=0.007; CIN 3 trend p=0.01). No similar trends were observed in CIN 2. Further details on the age and type-specific prevalence of HPV are provided in Additional file 1: Appendix Tables 1A and 2A.

### HPV prevalence in high grade lesions in New Zealand compared with Australia

The prevalence of any oncogenic HPV type in women with histologically-confirmed CIN 2+ was significantly higher in New Zealand compared with that reported in Australia (94.9% vs. 91.0%; p=0.04) (Table 4). HPV 16 was the most common type in both countries (51.2% in New Zealand and 51.4% in Australia). HPV 18 infection rates were not statistically significantly different between the countries (12.1% in New Zealand vs. 9.2% in Australia), although combined rates of infection for HPV16 and/or 18 appeared slightly higher in New Zealand (60.6% in New Zealand vs. 57.2% in Australia). Significantly higher reported prevalence of HPV types 52, 58 and 68 were observed in New Zealand compared with Australia (Table 4). The prevalence of type 52 was reported as 13.9% in Australia compared to 18.8% in New Zealand.

**Table 4:** Country and Regional comparison of oncogenic HPV in histologically-confirmed CIN 2, CIN 3 or CIS lesions

**HPV type**	New Zealand ^*^(N=356)			**Australia**^**† **^**(N=533)**			**Global regions**^‡^			
							**Sample weighted mean (%)**			
	**n**	**%**	**(95% CI)**	**n**	**%**	**(95% CI)**	**Asia**	**Europe**	**North America**	**South/Central America**
16	181	50.8	(45.5-56.2)	274	51.4	(47.1-5.7)	33.7	51.5	33.5	37.6
52^β^	67	18.8	(14.9-23.3)	74^§^	13.9	(11.1-7.1)	9.5	2.0	4.8	4.7
31	62	17.1	(13.4-21.5)	77	14.4	(11.617.7)	5.4	9.5	13.1	6.0
33	47	13.2	(9.9-17.2)	49	9.2	(6.9-12.0)	5.9	8.1	5.0	5.1
18	43	12.1	(8.9-15.9)	49	9.2	(6.9-12.0)	6.6	6.0	8.3	5.4
58^β^	40	11.5	(8.4-15.3)	37	6.9	(4.9-9.4)	12.2	3.5	6.8	11.2
51	36	10.1	(7.2-13.7)	61	11.4	(8.9-14.5)	5.1	2.0	3.4	4.0
39	27	7.3	(4.8-10.5)	44	8.3	(6.4-11.3)	1.2	1.4	3.7	2.4
35	18	4.8	(2.8-7.5)	24	4.5	(2.9-6.6)	3.3	2.2	2.8	3.9
45	16	4.5	(2.6-7.2)	23	4.3	(3.1-6.8)	0	1.5	2.3	6.0
59	15	4.2	(2.4-6.9)	26	4.9	(3.2-7.1)	2.3	0	1.9	1.0
56	10	2.8	(1.4-5.1)	28	5.3	(3.5-7.5)	3.6	2.9	8.7	2.1
68^β^	8	2.2	(1.0-4.4)	12	2.3	(1.2-3.9)	1.1	0.2	2.4	0.5
Any ^β^	342	94.9	(92.1-97)	485	91.0	(88.2-3.3)	78.0	87.3	78.8	77.9

Oncogenic HPV includes infection with either type 16, 18, 31, 33, 35, 39, 45, 51, 52, 56, 58, 59, or 68; CIN cervical intra-epithelial neoplasia; CIS carcinoma in situ.

* Limited to women with histologically-confirmed CIN 2/3. Excludes AIS, glandular dysplasia or cervical cancer. As discussed in the text, the population was ‘enriched’ because all were originally referred with high grade cytology.

*† From Stevens et al. (2009).*[9]*.This study included histologically-confirmed CIN 2+, but squamous cell carcinoma constituted only 9 of 533 cases.*

*‡ Re-calculated from data in*[24]*(see text). African region had no studies with HPV type-specific measurement in histologically-confirmed high grade disease.*

β Statistically significant difference in prevalence between New Zealand and Australia when compared using HPV prevalence in CIN 2 from both studies.

§ HPV type 52 detected via Linear Array in the absence of types 33, 35 or 58; or confirmed via an in-house probe designed to detect type 52 within this subgroup of co-infections. Results for type 52 alone not validated for linear array testing.

### HPV prevalence in high grade lesions in New Zealand compared with countries in other regions

Compared with other regions included in the worldwide meta-analysis [24], the prevalence of any oncogenic HPV type in women with histologically-confirmed CIN 2/3 (excluding women with cancer, AIS or glandular lesions) was greater in this ‘enriched’ New Zealand population (94.9%) compared with either Asia (78%), Europe (87.3%), North America (78.8%) or South/Central America (77.9%) (Table 4).

The prevalence of HPV 16 in CIN 2/3 in this enriched population of women was 50.8% (Table 4). This was similar to that observed in Europe (51.5%), but higher than Asia (33.7%), North America (33.5%) and South/Central America (37.6%). Similarly, HPV 18 appeared marginally higher in New Zealand (12.1%) compared with North America (8.3%), and was approximately twice as high as observed in Asia (6.0%), Europe (6.5%) and South/Central America (5.4%). The reported prevalence of HPV 52 was much higher than that reported compared with non-Oceania regions, and almost double that reported for Asia (18.8% vs. 9.5%).

## Discussion

This study is the first to estimate the prevalence of oncogenic HPV among women with high grade lesions in New Zealand. The findings of this survey confirm that, as for other regions, HPV 16 is the most common HPV type among women with a high grade cytology report and in women with histologically-confirmed CIN2+. The prevalence of HPV16 in CIN 2/3 in New Zealand was broadly consistent with that in Australia and Europe (about 50%) [9] but was higher than that reported for North America, Asia, and South/Central America (all less than 40%) [24]. In New Zealand, HPV 18 was observed in 12.1% of women with a histological diagnosis of CIN2/3. This was broadly consistent with reported rates in Australia and North America but more than that reported in Asia, Europe and South/Central America.

The study has also provided new more detailed information than has been reported to date on the pattern of the relative prevalence of HPV 16 and 18 infections in high grade lesions by age. For HPV type 16/18, prevalence peaked among women aged 20–29 years and fell with increasing age. Conversely, for the OHR HPV types, prevalence was lowest among women aged 20–29 years, and increased with increasing age to a peak among women aged 40–69 years. This observation is broadly consistent with patterns observed in the Guanacaste, Costa Rica cohort, which highlighted a similar pattern for HPV 16 and OHR types [25]. It is also consistent with the findings of a study of age-specific HPV prevalence in CIN 3/AIS as registered in 3 US cancer registries (Michigan, Iowa, California) between 1994–200 [26], with a study of the age-specific HPV prevalence in Danish women subsequently diagnosed with CIN2/3, [27] and with a study in United Kingdom which examined the prevalence of HPV 16/18 in histologically-confirmed CIN3 [17]. This age-related pattern of infection supports a proposed model of disease development that contends that HPV16 is more likely to progress to CIN3 precancerous disease within a shorter period, whereas OHR types progress slowly and less frequently to precancerous abnormalities [2].

An important strength of the study is that we recruited from the population-based National Cervical Screening Program register. A total of 27% of the population initially identified as potentially eligible were included in the final analysis, after taking into account exclusions, the ability to contact women, consent and HPV test success rates. However, of the women who could be approached a relatively high consent rate was obtained, and the observed distributions of age and ethnicity in the study population were very similar to that in the broader screening population of women aged 20–69 years with a reported high grade cytology result in New Zealand (Table 2). The extent of underlying high grade CIN2+ in the study population of women with high grade cytology (about 70% overall) was also very similar to that reported in the population overall for the first half of 2009 (70.2%) [15]. A similar distribution of oncogenic HPV types in women with histologically-confirmed CIN 2+ was observed in women recruited via both recruitment channels (Additional file 1: Appendix Table 3A). These findings suggest that the overall study results are broadly representative of the wider population of women with high grade cytology.

In our study, and in the prior Australian study, the overall prevalence of oncogenic HPV in CIN 2/3 appeared to be somewhat higher than reported other regions. One explanation for this finding is improvements in PCR technology used to detect and genotype HPV infection. In the period between studies reported in the worldwide meta-analysis and the current New Zealand study, PCR testing has undergone two significant improvements in technology: the development of new oligonucleotide primers (PGMY09/11) and the inclusion of an AmpliTaqGold polymerase. PGMY09/11 primers were designed using DNA sequence homology for various HPV types indicated within the highly conserved region of the L1 region. In contrast to this method of designing primers, the original ‘manos’ MY09/11 primers used in previous prevalence studies were ‘degenerate’, which meant they did not necessarily provide reproducible estimates of HPV types detectable in samples [20]. The improvement in design accompanying the PGMY primers translated into greater sensitivity for detecting HPV types 26, 35, 42, 45, 52, 54, 55, 59, 66, and 73 when compared with the traditional ‘manos’ MY09/11 primers [20]. In addition to improvements in primer design, AmpliTaqGold polyermase, introduced in early 2000, improved enzymatic characteristics and subsequent sensitivity for detecting HPV type infections compared with the older AmpliTaq polymerase [28]. Another factor that may account for the higher overall prevalence of oncogenic HPV in our study, relative to previous reports, is the method of recruitment. In this study, women were recruited following a high grade cytology report. We then assessed the prevalence of overall and type-specific oncogenic HPV in the subgroup of women with histologically-confirmed high grade disease. Our approach may have led to a more concentrated recruitment of women with ‘true’ high grade CIN. By contrast, previous studies may have been more likely to have included women with misclassified CIN 2 lesions [29]. Our clinical approach to enriching the study population provides a potential addition or alternative to implementing a recently proposed ‘gold standard’ for diagnosing CIN2/3 histology, involving use of laser capture microdissection to characterize the molecular features of suspected pre-cancerous lesions.

In the current study it was assumed that samples positive for type 52 alone represented true type 52 infection, but that samples testing positive for 52 and also for at least one of types 33, 35 and 58 were not truly positive for type 52 (i.e. it was assumed apparent type 52 reactivity represented cross-reactivity). Dedicated probes are required for accurate detection of HPV type 52 in the presence of multiple infections [9]. However, in practice, cross-reactivity was not a major concern in the current study since only one sample was positive for another type in addition to type 52. Because of our conservative approach to this aspect of the analysis, our finding of a high prevalence for type 52 may be, if anything, a slight underestimate.

This study provides a baseline measure of oncogenic HPV prevalence in a population of women in New Zealand, and provides a baseline for future similar surveys to assess the impact of HPV vaccination. Genotyping studies for high grade lesions have previously been included in a list of key recommendations to monitor the effect of HPV vaccination [30]. Our findings imply that the current HPV vaccination program in New Zealand, which involves delivery of a vaccine against HPV types 16/18, could prevent up to 62% of high grade lesions (53% of CIN 2 and 66% of CIN 3+). These estimates are based on the assumption (in the best case for vaccination) that co-infection of other oncogenic types, in the presence of HPV 16 and/or 18, was not causally responsible for the development of the majority of high grade lesions in the current study; if this assumption does not hold then the proportion of vaccine-preventable high grade lesions could be considerably lower. These estimates of potential vaccine effect should be considered highly provisional, and a number of other factors will be influential, including vaccine program coverage in New Zealand, prior expose of the catch-up cohort to vaccine-included HPV types, and the potential for cross-protection against non-vaccine-included types. Further surveillance of oncogenic HPV infection in histologically-confirmed high grade disease in the post-vaccination era in New Zealand will continue to be informative.

## Conclusions

The baseline assessment of oncogenic HPV prevalence in New Zealand women with high grade disease provides insight into the potential burden of disease avertable with HPV vaccination. The combined prevalence of oncogenic HPV 16 and 18 in New Zealand was found to be approximately 62% in confirmed high grade lesions. Ongoing surveillance of the prevalence of oncogenic HPV in high grade disease will allow future assessment of the impact of the national HPV immunization program in New Zealand.

## Competing interests

KC is co-PI of a new trial of primary HPV screening in Australia that is partially supported by Roche Molecular Diagnostics, Pleasanton, CA, USA. Other authors have no competing interests to declare. This study was funded by the New Zealand Ministry of Health.

## Authors’ contributions

LS participated in the development of the study protocol, undertook the analysis of the data and participated in drafting the manuscript. HL participated in the development of the study protocol, co-ordinated the study in New Zealand and participated in drafting the manuscript. MS participated in the development of study protocol and drafting of the manuscript. HN participated in the development of the study protocol, setting up the study at various sites and participated in the drafting of the manuscript. CB undertook the HPV genotyping and participated in the drafting of the manuscript. KC participated in the development of study protocol, participated in drafting the manuscript, and supervised the analysis and drafting of the manuscript. All authors read and approved the final manuscript.

## Pre-publication history

The pre-publication history for this paper can be accessed here:

http://www.biomedcentral.com/1471-2334/13/114/prepub

## Supplementary Material

Additional file 1: Appendix Figure 1A Recruitment process for women who provided a sample during colposcopy. **Figure 2A.** Recruitment process for women approached to provide consent to test after a high grade cytology result used to notify NCSP-R. **Table 1A.** Oncogenic HPV prevalence by age in high grade cytology and in histologically-confirmed CIN 2/3. **Table 2A.** Oncogenic HPV prevalence by age in histologically-confirmed disease, by subcategory (CIN2, CIN3 and CIN2/3 combined). **Table 3A.** Type-specific prevalence of oncogenic HPV in histologically-confirmed CIN2+ by sample collection method^**†**^. Click here for file
