# Supplementary material for: Type-specific oncogenic human papillomavirus infection in high grade cervical disease in New Zealand
Source: BMC Infect Dis. 2013 Mar 3;13:114. doi: 10.1186/1471-2334-13-114 (PMC3607885; doi:10.1186/1471-2334-13-114)
Supplement: Additional file 1: Appendix Figure 1A — Recruitment process for women who provided a sample during colposcopy. Figure 2A. Recruitment process for women approached to provide consent to test after a high grade cytology result used to notify NCSP-R. Table 1A. Oncogenic HPV prevalence by age in high grade cytology and in histologically-confirmed CIN 2/3. Table 2A. Oncogenic HPV prevalence by age in histologically-confirmed disease, by subcategory (CIN2, CIN3 and CIN2/3 combined). Table 3A. Type-specific prevalence of oncogenic HPV in histologically-confirmed CIN2+ by sample collection method†. [file 1471-2334-13-114-S1.doc]

# appendix

| **Figure A1. Recruitment process for women who provided a sample during colposcopy** |
| --- |
| *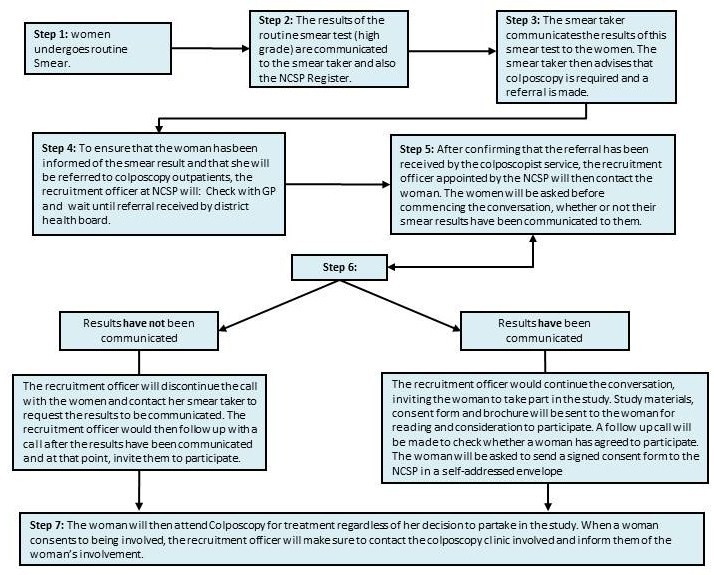* |

| Figure A2. Recruitment process for women approached to provide consent to test after a high grade cytology result used to notify NCSP-R |
| --- |
| 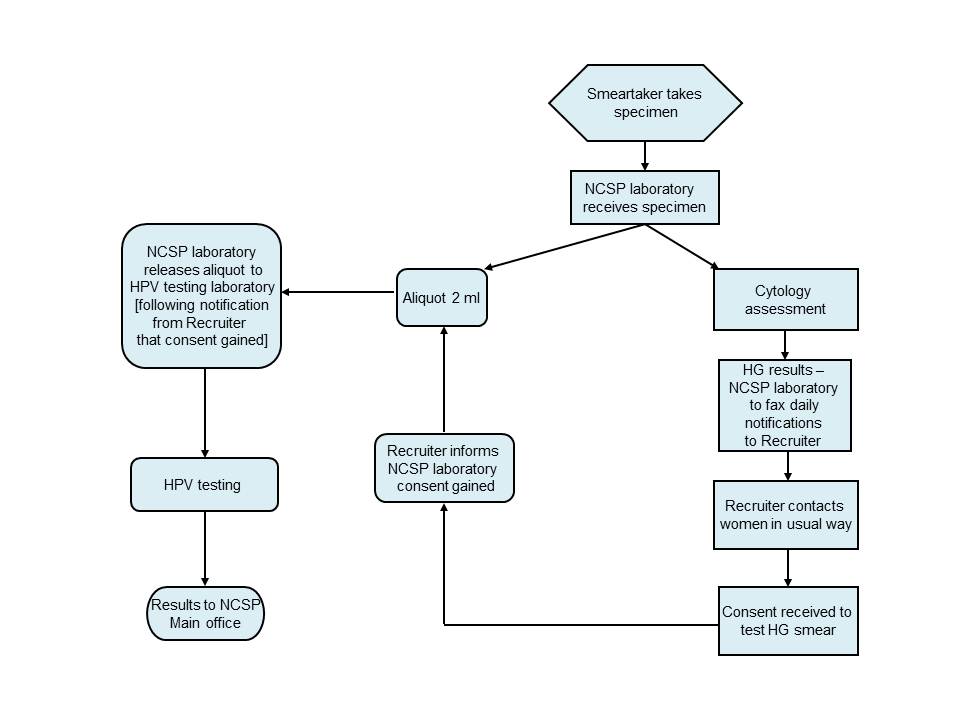 |

| Table A1. Oncogenic HPV prevalence by age in high grade cytology and in histologically-confirmed CIN 2/3 | | | | | | | | | | | |
| --- | --- | --- | --- | --- | --- | --- | --- | --- | --- | --- | --- |
| **Age** | **High grade cytology^*^** | **CIN 2/3^β^** | **Any 16and/or18** | | | **Other HR types (non 16/18)^‡^** | | | **Any HR types** | | |
|  |  |  | **HG cytology** | **CIN 2/3** | **p-value** | **HG cytology** | **CIN 2/3** | **p-value** | **HG cytology** | **CIN 2/3** | **p-value** |
|  | **N** | **N** | **% (95% CI)** | **% (95% CI)** |  | **% (95% CI)** | **% (95% CI)** |  | **% (95% CI)** | **% (95% CI)** |  |
| **20-29** | 284 | 177 | 62 (56.0-67.6) | 68.4 (61.0-75.1) | 0.16 | 32.4 (27.0-38.2) | 28.2 (21.7-35.5) | 0.35 | 94.4 (91-96.7) | 96.6 (92.8-98.7) | 0.27 |
| **30-39** | 171 | 116 | 50.9 (43.1-58.6) | 53.4 (44-62.8) | 0.67 | 35.1 (28.0-42.7) | 41.4 (32.3-50.9) | 0.28 | 85.4 (79.2-90.3) | 94.8 (89.1-98.1) | 0.01 |
| **40-69** | 139 | 63 | 36.7 (28.7-45.3) | 46.0 (33.4-59.1) | 0.21 | 35.3 (27.3-43.8) | 44.4 (31.9-57.5) | 0.21 | 74.1 (66-81.2) | 92.1 (82.4-97.4) | 0.003 |
| **20-69** | 594 | 356 | 52.9 (48.8-56.9) | 59.8 (54.5-65) | 0.04 | 33.8 (30.0-37.8) | 35.4 (30.4-40.6) | 0.63 | 87.0 (84.1-89.6) | 95.2 (92.5-97.2) | <0.0001 |
| *HR HPV high risk human papillomavirus; HG high grade; CIN cervical intra-epithelial neoplasia.*  ** High grade cytology refers to women with a reported index screening round cytology result of either high grade squamous intra-epithelial lesions (HSIL), atypical squamous cells, cannot rule out a high grade lesion (ASC-H), atypical glandular cells(AGC) or adenocarcinoma in situ (AIS).*  *‡ Other HR types includes infection with either type 31, 33, 35, 39, 45, 51, 52, 56, 58, 59 or 68.*  ***^β^*** *Excludes AIS, glandular dysplasia or cervical cancer.* | | | | | | | | | | | |

| Table A2. Oncogenic HPV prevalence by age in histologically-confirmed disease, by subcategory (CIN2, CIN3 and CIN2/3 combined) | | | | | | | | | | | | |
| --- | --- | --- | --- | --- | --- | --- | --- | --- | --- | --- | --- | --- |
| **Age group** | **CIN 2/3 combined^†^** | | | | **CIN 2^†^** | | | | **CIN 3^†^** | | | |
|  |  | **16 and/or 18** | **OHR**^‡^ | **Any HR** |  | **16 and/or 18** | **OHR**^‡^ | **Any HR** |  | **16 and/or 18** | **OHR**^‡^ | **Any HR** |
|  | **N** | **%** | **%** | **%** | **N** | **%** | **%** | **%** | **N** | **%** | **%** | **%** |
|  |  | ***(95% CI)*** | ***(95% CI)*** | ***(95% CI)*** |  | ***(95% CI)*** | ***(95% CI)*** | ***(95% CI)*** |  | ***(95% CI)*** | ***(95% CI)*** | ***(95% CI)*** |
| **20-29** | 177 | 68.4 | 28.2 | 96.6 | 65 | 58.5 | 38.5 | 96.9 | 112 | 74.1 | 22.3 | 96.4 |
|  |  | *(61.0-75.1)* | *(21.7-35.5)* | *(92.8-98.7)* |  | *(45.6-70.6)* | *(26.7-51.4)* | *(89.3-99.6)* |  | *(65.0-81.9)* | *(15.0-31.2)* | *(91.1-99)* |
| **30-39** | 116 | 53.4 | 41.4 | 95 | 52 | 50.0 | 46.2 | 96.2 | 64 | 56.3 | 37.5 | 93.8 |
|  |  | *(44.0-62.8)* | *(32.3-50.9)* | *(89.1-98.1)* |  | *(35.8-64.2)* | *(32.2-60.5)* | *(86.8-99.5)* |  | *(43.3-68.6)* | *(25.7-50.5)* | *(84.8-98.3)* |
| **40-69** | 63 | 46.0 | 44.4 | 92.1 | 35 | 45.7 | 45.7 | 94.3 | 28 | 46.4 | 42.9 | 89.3 |
|  |  | *(33.4-59.1)* | *(31.9-57.5)* | *(82.4-97.4)* |  | *(28.8-63.4)* | *(28.8-63.4)* | *(80.8-99.3)* |  | *(27.5-66.1)* | *(66.1-12)* | *(71.8-97.7)* |
| **20-69** | 356 | 59.6 | 35.4 | 95.2 | 152 | 52.6 | 42.8 | 96.1 | 204 | 64.7 | 29.9 | 94.6 |
|  |  | *(54.5-65)* | *(30.4-40.6)* | *(92.5-97.2)* |  | *(44.4-60.8)* | *(34.8-51)* | *(91.6-98.5)* |  | *(58.2-71.7)* | *(23.7-36.7)* | *(90.6-97.3)* |
| **Test for trend** |  |  |  |  |  |  |  |  |  |  |  |  |
| *chi-square* |  | 11.83 (1 d.f) | 7.21 (1 d.f) | 2.13 (1 d.f) |  | 1.63 (1.d.f) | 0.63 (1 d.f) | 0.39 (1 d.f) |  | 10.12 (1.d.f) | 6.6 (1 d.f) | 2.31 (1 d.f) |
| *p-value* |  | 0.0006 | 0.007 | 0.14 |  | 0.2 | 0.43 | 0.53 |  | 0.002 | 0.01 | 0.07 |
| *HR high risk; HPV human papillomavirus; CIN cervical intra-epithelial neoplasia*  ** HR HPV includes infection with either type 16, 18, 31, 33, 35 39, 45, 51, 52, 56, 58, 59 or 68.*  *† Excludes AIS, glandular dysplasia or cervical cancer.*  *‡Other HR types includes infection with either type 31, 33, 35 39, 45, 51, 52, 56, 58, 59 or 68, not 16 or 18.* | | | | | | | | | | | | |

*Differences in the prevalence of HR HPV type by recruitment method*

| **Table A3. Type-specific prevalence of oncogenic HPV in histologically-confirmed CIN2+ by sample collection method^†^** | | | | | | | | |
| --- | --- | --- | --- | --- | --- | --- | --- | --- |
|  | **At colposcopy (N=138)** | | | | **Residual cytology material (N=232)** | | | |
| **HR HPV type** | **n** | **%** | **95% CI** | | **n** | **%** | **95% CI** | |
| 16 | 66 | 47.8 | 39.3 | 56.5 | 123 | 53.0 | 46.4 | 59.6 |
| 52 **^β^** | 24 | 17.4 | 11.5 | 24.8 | 45 | 19.4 | 14.5 | 25.1 |
| 31 | 25 | 18.1 | 12.1 | 25.6 | 37 | 15.9 | 11.5 | 21.3 |
| 33 | 23 | 16.7 | 10.9 | 24.0 | 24 | 10.3 | 6.7 | 15.0 |
| 18 | 15 | 10.9 | 6.2 | 17.3 | 33 | 14.2 | 10.0 | 19.4 |
| 58 | 19 | 13.8 | 8.5 | 20.7 | 23 | 9.9 | 6.4 | 14.5 |
| 51 | 14 | 10.1 | 5.7 | 16.4 | 21 | 9.1 | 5.7 | 13.5 |
| 39 | 13 | 9.4 | 5.1 | 15.6 | 13 | 5.6 | 3.0 | 9.4 |
| 45 | 7 | 5.1 | 2.1 | 10.2 | 10 | 4.3 | 2.1 | 7.8 |
| 59 | 4 | 2.9 | 0.8 | 7.3 | 13 | 5.6 | 3.0 | 9.4 |
| 35 | 8 | 5.8 | 2.5 | 11.1 | 8 | 3.4 | 1.5 | 6.7 |
| 56 | 4 | 2.9 | 0.8 | 7.3 | 6 | 2.6 | 1.0 | 5.5 |
| 68 | 5 | 3.6 | 1.2 | 8.3 | 3 | 1.3 | 0.3 | 3.7 |
| 16 and/or 18 | 76 | 55.1 | 46.4 | 63.5 | 149 | 64.2 | 57.7 | 70.4 |
| OHR* | 56 | 40.6 | 32.3 | 49.3 | 70 | 30.2 | 24.3 | 36.5 |
| Any HR HPV | 132 | 95.7 | 90.8 | 98.4 | 219 | 94.4 | 90.6 | 97.0 |
| *† Comparison excludes 5 study participants with CIN2+ due to their unknown source of recruitment*  *β Calculated as infection with HPV type 52 alone.*  **chi-square statistic p value <0.05 when HPV prevalence is compared between the two recruitment arms.* | | | | | | | | |
